# In vivo cartilage tissue engineering

**DOI:** 10.1186/s13018-018-0823-0

**Published:** 2018-05-08

**Authors:** B. Gurer, S. Cabuk, O. Karakus, N. Yilmaz, C. Yilmaz

**Affiliations:** 1 Mersin University Medical School, Mersin, Turkey; 2 Omer Halis Demir University Hospital, Nigde, Turkey; 3 Department of Orthopedics and Traumatology, Mersin University Medical School, Mersin, Turkey; 4 Fatih Sultan Mehmet Teaching and Research Hospital, İstanbul, Turkey; 5 Department of Histology and Embryology, Mersin University Medical School, Mersin, Turkey; 6 İstanbul, Turkey

**Keywords:** Catilage matrix protein, Collagen-induced, Cartilage diseases, Knee injuries

## Abstract

**Background:**

Biologic treatment options for cartilage injuries require chondrocyte expansion using cell culture. Clinical application is accomplished in two surgical sessions and is expensive. If isolation of chondrocytes and stimulus for proliferation and extracellular matrix synthesis can be achieved in vivo, the treatment can be performed in one session and the cost can be reduced.

**Methods:**

A 2.5-cm diameter full-thickness chondral defect was created in the knees of five groups of sheep. In one group, some of the chondral tissues obtained from the creation of the defect were diced into small pieces and were placed into the defect and were covered with a collagen membrane (MIV group). In the other group, the collagen membrane was soaked in collagenase prior to usage. In the next group, the collagen membrane was soaked in both collagenase and growth factors. Matrix-induced autologous chondrocyte implantation (MACI) was applied to another group in two sessions, and the last group was left untreated. After 15 weeks of follow-up, repair tissues were compared macroscopically, histomorphometrically, and biochemically for tissue concentrations of glycosaminoglycan and type II collagen.

**Results:**

MACI and MIV groups demonstrated better healing than others and were similar. Addition of collagenase or growth factors did not improve the results. Addition of collagenase did not have detrimental effect on the surrounding cartilage.

**Conclusions:**

With the described method, it is possible to obtain comparable results with MACI. Further studies are also needed to see if it works similarly in humans.

## Background

Large chondral defects require special attention. Autologous chondrocyte implantation (ACI) was described as a viable solution for large chondral defects. But the technique has its disadvantages. Patients undergo two operations, and the second one is an open surgery. Fixation of the periosteal graft over the defect is technically difficult and prone to failure. Cells in suspension tend to gather at edges rather than distributing homogenously into the volume of the defect. Matrix-induced autologous chondrocyte implantation (MACI) was described in 1999 to overcome most of these disadvantages and as of 2002 was accepted as the second generation of ACI [1, 2]. Although it does overcome most disadvantages of the aforementioned technique, MACI still requires two surgeries and is expensive. Both of these modalities utilize cell culture techniques. The cell culture technique depends on isolating mature chondrocytes from their extracellular matrix, forcing them to convert into a proliferative phase. Mature chondrocytes which provide continuity of the extracellular matrix, when isolated from their lacunae, under suitable conditions, start to proliferate. The suitable conditions are provided by the incubator. Incubator mimics in vivo environment. But the environmental variables, such as temperature, humidity, CO_2_ and O_2_ concentrations, and nutrition are readily available in ideal amounts in vivo. We thought that if the chondrocytes could be isolated inside the body, forced to transform to the proliferative phase and kept in the defective area, the treatment could be accomplished in one session.

## Methods

The experiment was conducted with the permission of the institutional ethics committee. Nineteen merino female sheep aging between one and two were used as subjects. The subjects were numbered using ear tags and were grouped as follows:

- Four sheep as “MACI in vivo” (MIV) group,
- Four sheep as MIV + collagenase group,
- Four sheep as MIV + collagenase + growth factors (GF) group,
- Four sheep as MACI group, and
- Three sheep as control group.

The subjects were kept in a private fold with unlimited access to food and water during the study.

All subjects received subcutaneous injection of 7.5% 10 mL cefquinom 30 min prior to surgery, and the antibiotic prophylaxis was carried on for three days. Induction of anesthesia was provided by 0.3 mg/kg intramuscular xylazine followed by 2 mg of ketamine injection. The right knees of all sheep were shaved, and medial patellar arthrotomy was done via midpatellar skin incision. A circle of 2.5 cm in diameter (approximately 5 cm^2^) was drawn on the central trochlear cartilage, and a chondral defect was created with the help of scalpel and curette. Cartilage including the calcified layer was removed, and depth of the defect reached the subchondral bone. The procedure was terminated at this level for the control and the MACI group subjects, and the surgical incisions were sutured.

For the MIV, MIV + collagenase, and MIV + collagenase + GF groups, 140 to 200 mg of the cartilage tissue obtained from the formation of the defect was weighed and diced into 1 mm^3^ pieces making up 10 to 12 pieces. Base of the defect was covered with fibrin glue (Tisseel, Baxter, USA), and the cartilage tissue pieces were placed into the defect homogenously. A type I/III collagen membrane (Chondrogide, Geistlich Pharma, Wolhusen, Switzerland) was tailored matching the size and shape of the defect, and the cartilage pieces were covered with this membrane with the rough side facing the defect and the smooth side facing outside. In the MIV group, the membrane was used as is. In the MIV + collagenase group, the membrane was soaked into 0.2% type II collagenase solution (Biochrom AG, Berlin, Germany) for 30 min prior to application. In the MIV + collagenase + GF group growth factors were added to the collagenase solution. As growth factors Insulin (1 g/L) – Transferrin (0.55 g/L) – Selenium (0.00067 g/L) (ITS solution 1% (*v*/*v*), Gibco, Invitrogen, NY) and ascorbic acid 50 μg/mL (0.4 mL solution, Gibco, Invitrogen, NY) were used. Patella was relocated, and the knee was flexed and extended repeatedly to test stability of the membrane and the capsule. Subcutaneous tissue and the skin were sutured.

One hundred forty to 200 mg of the chondral tissue obtained from the creation of the defects in the MACI group were diced into 1 mm^3^ pieces and placed into tissue transfer solution (Dulbecco’s modified Eagle medium—DMEM). In the laboratory, the tissue pieces were rinsed with sterile PBS and placed into 15 mL Falcon tubes containing Dulbecco’s modified Eagle medium (DMEM, 10% Fetal Bovine Serum, 1% penicillin – streptomycin, 1% amphotericin B, 2.5% L-glutamine (Biochrom AG, Berlin, Germany)). They were kept in 0.2% type II collagenase (Biochrom AG, Berlin, Germany) for 48 h, and precipitate from each piece was placed in a culture flask. Culture flasks containing DMEM were incubated at 37 °C and 5% CO_2_. Nutrition was provided by changing the medium every 3 to 4 days until the cells reached confluence. The cells were mobilized by trypsin. Viability was confirmed by trypan blue dye exclusion test, and the suspension was centrifuged to form pellets of cells. Pellets were collected and seeded on the rough face of precut 2.5 cm diameter type I/III collagen membrane (Chondrogide, Geistlich Pharma, Wolhusen, Switzerland). The implant was incubated for another 72 to 96 h. The approximate number of cells per membrane was 2 × 10^6^. The cell expansion process took approximately 6 weeks. At the end of this time, the MACI group sheeps were prepared for the second surgery similar to the first one. Previous incision scar was used and the defect was debrided. Any possible bleeding from the subchondral bone was stopped by adrenaline-soaked gauze compression. The prepared MACI implant was glued to the base of the defect with Tisseel (Baxter, IL, USA) fibrin glue. Rough face of the collagen membrane on which the chondrocytes were seeded faced the defect and the smooth face faced outside. Stability of the construct was tested through repeated flexion and extension, and the incisions were closed.

All subjects received 8 cm^3^ of intramuscular metamizole for postoperative analgesia. To prevent weight bearing, a tennis ball was wired to the operated extremity hoof. The tennis balls were removed 6 weeks after the index operation. At the end of the 15th week (for MACI group, 15 weeks after the second operation), all subjects were sacrificed and both knees were excised.

The defective cartilage area was first assessed macroscopically. A scoring system recommended by Rudert et al. was used [3] (Table 1). Scoring was performed by two researchers (CY, SC) blindly and the averages of the two results were recorded.

**Table 1:** Scoring system for macroscopic assessment of cartilage healing [3]

Criteria	Score	Macroscopical properties
Filling	1	Significantly below adjacent cartilage level
	2	Same level with adjacent cartilage, central depression
	3	Same level with adjacent cartilage
Color	1	Brown or yellow
	2	White
	3	Same as adjacent cartilage
Surface	1	Rough
	2	Smooth

For further evaluation, whole defect areas including the subchondral bone were excised. A 1 × 1-cm chondral sample from the patella opposite to the defect area and another similar-sized sample from the contralateral knee’s trochlear region corresponding to the defect area were obtained. Half of all the samples were cut for histological examination, and the other halves were frozen at − 80 °C for biochemical evaluation.

The samples saved for histological evaluation were placed into 10% neutral formaldehyde containing jars and kept at least 48 h for fixation. After fixation, the tissues were decalcified using a commercial nitric acid and formic acid mixture for 3 to 5 days. Following decalcification, tissue samples were passed through alcohol, xylol, and were embedded in paraffin respectively. The tissues were sliced into ten equal parts, and 5-μm thick sections were taken from each slice. Sections were stained with hematoxylin eosin and safranin-o fast green and examined under Olympus BX50 light microscope (Olympus GmBH, Germany) and were photographed using Nikon Coolpix 5000 digital camera (Nikon Corp., Tokyo, Japan) attached to the microscope. Sections were scored by two blinded residents according to the modified O’Driscoll Scoring System, and the average score of the ten sections were recorded (Table 2).

**Table 2:** Modified O’Driscoll Scoring System [36, 37]

Category	Score
1. Nature of predominant tissue	
Cellular morphology	
Hyaline articular cartilage	4
Partially differentiated mesenchyme	2
Fibrous tissue or bone	0
2. Structural properties	
Surface integrity	
Smooth and in tact	3
Superficial horizontal lamination	2
Fissures: 25–100% of thickness	1
Significant disruption, including fibrillation	0
Structural integrity	
Normal	2
Slight disruption, including cysts	1
Significant disruption	0
Thickness	
100% of normal surrounding cartilage	2
50–100% of normal cartilage	1
0–50% of normal cartilage	0
Bonding at adjacent cartilage	
Bonded at both ends	2
Bonded at one end or partially at both	1
Not bonded	0
3. Independence from cellular changes of degeneration	
Hypocellularity	
Normal cellularity	3
Mild hypocellularity	2
Moderate hypocellularity	1
Severe hypocellularity	0
Chondrocyte grouping	
No grouping	2
Less than 25% of cells	1
25–100% of cells	0
4. Independence from degenerative changes of adjacent cartilage	
Cellular properties	
Normal cellularity, no grouping	3
Normal cellularity, mild grouping	2
Mild or moderate hypocellularity	1
Severe hypocellularity	0
Fibrillation	
No fibrillation	3
Less than 25% of the thickness of cartilage	2
25–50% of the thickness of cartilage	1
More than 50% of the thickness of cartilage	0

The samples saved for biochemical ELISA (enzyme-linked immuno sorbent assay) determination of glycosaminoglycan (GAG) and type II collagen concentrations were thawed overnight. Tissues were divided into two equal weight pieces. One piece from each sample was reserved for GAG analysis. Fifty milligrams of tissue was immersed into 1 mL of extraction buffer (pH 6.4 0.2 M sodium phosphate buffer including 15 mg of papain) and incubated at 65 °C overnight. Samples were centrifuged at 10,000 rpm for 10 min, and the supernatant was reserved. Concentration of tissue sulphate GAG (sGAG) was determined using Blyscan Sulphate Glycosaminoglycan Assay Kit (CUSABIO ELISA, Wuhan, China) quantitative dye-binding technique. One hundred microliters of the sample and standards were pipetted into the tubes and were stirred for 30 min in mechanical shaker with 1 mL of Blyscan dye reactive. This resulted in sGAG – dye complex. The tubes were centrifuged at 12,000 rpm for 10 min, and the supernatant was discarded. To dissociate the bonded dye, 0.5 mL of dissociation reactive was added onto the remaining pellets and stirred in vortex. Two hundred microliter of the solution was taken on plates, and absorbance under 656 nm was recorded. Concentrations were calculated using the standard curves.

The other half of the tissues were prepared for biochemical evaluation of type II collagen concentration. One hundred milligrams of tissue was homogenized in 1 mL of pH = 7.4 phosphate buffer and was kept at − 20 °C overnight for cell membrane degradation. The homogenate was centrifuged at 5000 rpm for 5 min, and the supernatant was acquired for the assay. Collagen type II ELISA kit (CUSABIO ELISA, Wuhan, China), which is formed of specific antibody covered pits, was used. Samples and standards were pipetted into the pits, and the plate was washed to remove free material. Type II collagen-specific biotin attached antibody was added and washed again. Avidin-conjugated horseradish peroxidase was added and washed. Addition of substrate solution followed by stop solution resulted in color change, and the color intensity was measured under 450 nm.

Macroscopic assessment scores, histomorphometric scores, and GAG and type II collagen concentrations of the five groups were compared statistically. Distribution similarity was determined using Kruskal-Wallis method, and the differing group was determined by Mann-Whitney *U* method. Bonferroni adjustment was used to keep significance level at 0.05. For within-group comparisons, Wilcoxon signed-rank test was used. Statistical analysis was accomplished using SPSS (SPSS 16.0, Chicago, IL, USA) software. *P* values lower than 0.05 were considered significant.

## Results

During the follow-up period, one sheep from groups MIV, MIV + collagenase + GF, and MACI died decreasing the total number of subjects from 19 to 16. The macroscopical and histomorphometric scores and tissue GAG and type II collagen concentrations were as listed in Table 3.

**Table 3:** Summary of the results

	MIV				MIV + collagenase					MIV + collagenase + GF				MACI				Control			
Subject No	1	2	3	AVG^10^	5	6	7	8	AVG	9	11	12	AVG	14	15	16	AVG	17	18	19	AVG
Macroscopic score	8	7	4	6	6	5	5	5	5	3	5	3	4	7	5	4	5	3	3	3	3
Histologic score: lesion^1^	10	8	8	9	6	8	7	7	7	8	8	7	8	10	9	8	9	2	2	2	2
Histologic score: intact^2^	24	24	24	24	24	24	24	24	24	24	24	24	24	24	24	24	24	24	24	24	24
Histologic score: patella^3^	24	24	24	24	24	24	24	24	24	24	24	24	24	24	24	24	24	24	24	24	24
GAG lesion^4^	11.5	11.7	11.3	11.50	11.0	12.2	11.6	10.9	11.43	10.8	9.9	7.6	9.43	11.8	12.1	11.9	11.93	10.4	10.5	10.4	10.43
GAG intact^5^	9.9	10.7	10.1	10.23	9.5	10.0	12.6	10.9	10.75	9.8	11.3	9.7	10.27	10.2	10.3	9.9	10.13	11.7	11.4	11.8	11.63
GAG patella^6^	11.0	10.9	10.8	10.90	11.5	11.9	10.8	11.0	11.30	11.8	10.6	11.9	11.43	11.6	11.0	11.8	11.47	11.5	11.4	11.4	11.43
Collagen type II: lesion^7^	22.5	34.7	20.6	25.93	9.2	10.0	8.6	8.0	8.95	7.5	6.7	6.4	6.87	41.8	40.0	41.5	41.10	2.5	2.4	2.5	2.47
Collagen type II: intact^8^	9.0	9.3	7.3	8.53	12.6	6.7	6.7	8.9	8.73	11.3	6.3	8.8	8.80	6.7	6.2	9.4	7.43	8.3	8.5	8.0	8.27
Collagen type II: patella^9^	8.4	7.9	7.8	8.03	8.9	9.2	7.7	8.6	8.6	7.9	8.6	9.1	8.53	8.	7.8	8.2	8.30	8.5	8.6	8.8	8.63

^1^Histomorphometric score of the experimental lesion area

^2^Histomorphometric score of the intact cartilage obtained from the contralateral knee

^3^Histomorphometric score of the patellar cartilage obtained from the ipsilateral knee

^4^Experimental lesion area tissue glycosaminoglycan concentration (μg/mL)

^5^Intact cartilage obtained from the contralateral knee tissue glycosaminoglycan concentration (μg/mL)

^6^Patellar cartilage obtained from the ipsilateral knee tissue glycosaminoglycan concentration (μg/mL)

^7^Experimental lesion area tissue type II collagen concentration (ng/mL)

^8^Intact cartilage obtained from the contralateral knee tissue type II collagen concentration (ng/mL)

^9^Patellar cartilage obtained from the ipsilateral knee tissue type II collagen concentration (ng/mL)

^10^Group average value

**Fig. 1:**
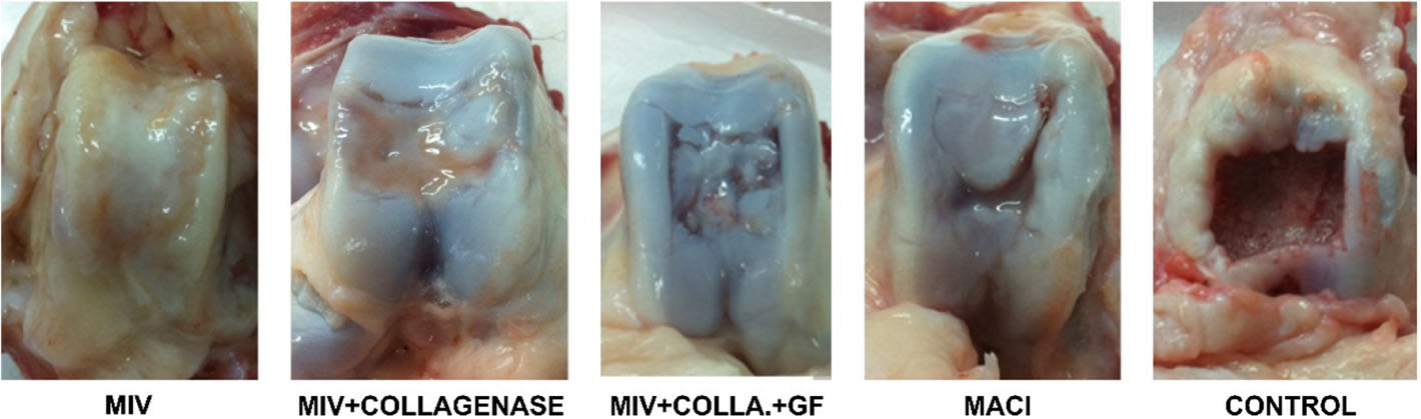
Macroscopic sight of the samples. Control sample shows no healing. Other groups demonstrate some extent of healing with the MIV, MIV + collagenase, and MACI groups scoring better than the GF added group

Macroscopically, the control group did not show any observable healing. MIV, MIV + collagenase, and MACI groups scored better than the control and the GF added groups, but the difference was not statistically significant (Fig. 1). The evaluation was done by two researchers, and the interobserver reliability kappa value was calculated as 0.84.

Distribution analysis yielded difference in histomorphometric scores (*p* = 0.018), GAG (*p* = 0.018), and type II collagen (*p* = 0.006) concentrations. The origin of difference was further analyzed by paired comparisons.

**Fig. 2:**
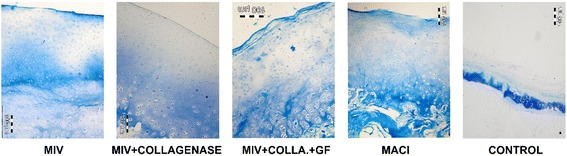
Safranin O fast green dyed, × 60 magnified histologic images of the samples. Control group demonstrates scarce healing tissue. MIV and MIV + collagenase groups demonstrate mature columnar cartilage structure. The well-formed cartilage structure seen on the MIV + collagenase group was not homogenous throughout the lesion but was in forms of islets. In contrast, the structure seen on the picture was homogenous throughout the lesion area in the MIV group. MIV + collagenase + GF group has a less organized structure with remnants of the collagen membrane on the surface. The MACI group shows less organized but satisfactorily thick healing. The structure was homogenous throughout the lesion area

Histomorphometrically, MIV group shared the highest average score (score = 9) with the MACI group (score = 9). The score was significantly higher than the control (*p* = 0.034) group, but the difference between MIV and MACI groups and the other two groups was not significant (*p* = 0.064 and *p* = 0.197). The other two treatment groups also had better scores compared to the untreated control group (*p* = 0.026 for the MIV + collagenase group, *p* = 0.034 for the MIV+ collagenase + GF group) (Fig. 2). When the samples from the lesion sites and the samples from the intact cartilage sites were compared within the groups, despite frank inferior scores, a valid statistic comparison could not be done due to the small sample size. The scoring procedure was done by two blinded residents, and the interobserver reliability kappa value was calculated as 0.84.

The highest *tissue GAG concentration* was measured in the MACI, MIV, and the MIV + collagenase groups. The difference between these groups and the control group was significant (*p* = 0.046, *p* = 0.046, *p* = 0.032 respectively). The GAG concentration in the MIV + collagenase + GF group was measured even lower than the control group (not reaching statistical significance). GAG tissue concentration did not differ between MACI, MIV, and MIV+ collagenase groups. Although the lesion GAG concentrations were higher than the intact cartilage sample GAG concentrations of the same animal (tissue from ipsilateral patella and the contralateral femoral groove), a valid statistic comparison for the difference could not be done because of the small size of the sample.

Highest *tissue type II collagen concentrations* were measured in the MACI group. The concentration was significantly higher than all groups (*p* = 0.046 for the control, *p* = 0.034 for MIV + collagenase, and *p* = 0.034 for MIV + collagenase + GF groups) but the MIV group (*p* = 0.05). In the MIV group, the concentration was significantly higher (*p* = 0.046) than that of the control group, and significantly higher than the collagenase added groups (*p* = 0.034). In the MIV + collagenase group, type II collagen concentrations were significantly higher than the control group (*p* = 0.032). But addition of the collagenase should have affected adversely because the type II collagen concentrations were significantly lower than the MACI and the MIV groups (*p* = 0.034 for both). Addition of growth factors to the implant did not also improve the results. Tissue type II collagen concentration was significantly higher than the control group (*p* = 0.046) but significantly lower than the other groups (*p* = 0.034). In the control group, the type II collagen concentration of the lesion was three to four times lower than that of the intact cartilage from the same animal. In the MACI group, the average concentration in the lesion was approximately five times more than that of the intact cartilage samples. In the MIV group, the collagen concentration was three times more than that of intact cartilage. In the MIV + collagenase group, the lesion and the intact cartilage contained similar amounts of collagen. In the GF added group, the collagen concentration was lower than the intact cartilage. The comparisons with the intact cartilage of the same animal did not yield valid statistical results because of the sample size.

Cartilage samples obtained from the ipsilateral patella and the contralateral femoral groove were compared macroscopically, histomorphometrically, and biochemically. No difference could be detected in structure, ultrastructure, thickness, and tissue concentrations of GAG and type II collagen.

## Discussion

Rabbits are the most commonly used experimental subjects in studies about cartilage healing. Tissue engineering studies require larger defects and abundant amount of cartilage tissue. International Cartilage Research Society recommends goat as cartilage defect model [4]. Similar weight sheep have larger knees than goats, and their knees resemble more to human knee in size, structure, and healing capacity aspects [4, 5]. Thus, sheep were the preferred experimental model in this study. Defects with a diameter less than 3 mm tend to heal spontaneously [6, 7]. Spontaneous healing limit increases up to 6 mm in larger animals [8]. It is known that defects with diameters less than 6 mm heal spontaneously in sheep [9, 10]. The defects created in this study were 25 mm in diameter. Lack of spontaneous healing was confirmed by the control group. ACI and MACI are indicated for defects over 2 cm^2^ in area [11]. Within the 2 to 5 cm^2^ range, osteochondral autogenous transfer (OAT) is an alternative treatment [12]. But over 5 cm^2^ OAT is not recommended, and the ACI and MACI remain the only solution options. We thus preferred to make the defect size about 5 cm^2^. Defect can either be placed on the distal femoral condyles or the trochlear groove. A 25-mm diameter defect does not fit into the distal femoral condyle of a sheep, and thus, we created the defects at the trochlear groove. Although susceptible to shear stresses, the trochlear groove provides a flat, wide area suitable for experimental studies [13].

Creation of the chondral defect yields healthy cartilage tissue. Instead of gathering from elsewhere, these cartilage tissues were used as donor tissues. For MACI, in order to proliferate sufficient amount of chondrocytes, 140 to 200 mg of cartilage tissue is required [14–18]. In this experiment, to simulate the clinical scenario and to be able to compare the methods, equal amount of the cartilage tissue was used for both MACI and MIV groups. The remaining tissues were discarded. MACI was done in two surgical sessions and MIV groups in one session also to mimic clinical scenario. One might think that it would be better to create the defects in the first session and do all the treatment procedures, including the MIV groups, in a second session so that it would resemble more to the clinical scenario of injury and treatment. Since, during the treatment procedures, the defect area is debrided and is converted to a fresh chondral injury, we chose to do the procedure in the same setting to shorten the follow-up time.

In the literature, most studies on rabbits did not utilize any kind of immobilization [19–22]. For dogs either external fixation or plaster cast immobilization was used [23, 24]. Sheep mobilization was usually limited by narrowing down fold size [25, 26]. We used a previously successful method to limit bearing weight while allowing mobilization and joint motion. In this method, authors fixed a tennis ball under the operated extremity nail to prevent the animal to bear weight with that extremity and protected repaired tendons for 6 weeks [27].

Original description of MACI included the use of a double-layered type I and III collagen membrane [28]. The rough side of the membrane has a loose structure and provides a suitable surface for the cells to attach, proliferate, and produce matrix. The smooth side of the membrane has a dense structure and while allowing nutrition exchange, prevents cell loss. In this study, we used the membrane which was used to produce the original MACI implant. Other substances which would provide a better 3D homogenous distribution of the cartilage tissues could also have been used, but for this study, we tried to keep the variables to a minimum.

The major event which forces mature chondrocytes to turn into proliferative phase chondrocytes is the removal of the matrix [29]. In cell culture laboratories, this is accomplished by enzymes like type II collagenase. The enzyme denatures collagen, and the chondrocytes are freed from the surrounding matrix. If proper conditions are provided, cells start to proliferate until they reach a confluence at the flask surface. The proper conditions are readily available in vivo. The major problem is to isolate chondrocytes from their matrix. In this study, we soaked the type I/III collagen membrane in type II collagenase solution. We wanted to see if the collagenase would act on the tissue pieces, digest collagen, and isolate chondrocytes, and to see if the isolated chondrocytes would proliferate and produce matrix. Since one of the most successful techniques in managing large chondral defects is the MACI, we preferred to compare the results to this method [30, 31]. As expected, the control group did not show any noteworthy healing. Within the treatment groups, the best results were obtained in the MACI group. MIV group, in which the cartilage tissue pieces were only covered with the collagen membrane, showed comparable healing. Macroscopically and histomorphometrically, MACI and MIV groups were similar. When the healing tissue GAG concentrations were compared, both MACI and MIV groups had similar and more GAG concentrations than the intact cartilage tissue. The difference was not statistically significant, but because of the limited sample size, statistical comparisons were not very reliable. Type II collagen concentrations in the MACI and the MIV groups were significantly more (3 to 6 times more) than that of the intact cartilage. Although it was more in the MACI group, this difference was again not statistically significant. Addition of collagenase did not yield the expected effect. Healing was worse macroscopically and histomorphometrically. Although healing tissue GAG concentration was similar to the other groups and the intact cartilage, type II collagen concentration of the tissue dropped significantly. We believe the addition of collagenase affected the newly formed collagen also. In this group, the samples obtained from the patella, which was in contact with the other side of the same membrane, did not differ from the samples obtained from the contralateral knee. We may say that addition of collagenase did not have a short-term detrimental effect on the other parts of the cartilage tissue.

Growth factors are widely used to promote and speed up proliferation of chondrocytes in cell cultures. The mostly used growth factor solution is ITS + ascorbic acid which consists of insulin, transferrin, selenium, and ascorbic acid. The concentrations recommended for cell culture were used in the study [32, 33]. The growth factor-added group gave the worst results in the study. Both GAG and type II collagen concentrations dropped below the other study groups and the intact cartilage levels. One or combination of the reagents would have caused this effect, and exact mechanism needs further investigation.

## Conclusions

Cartilage healing takes time. Follow-up time of experimental studies on cartilage healing varies between 6 to 52 weeks [8, 13, 23, 25, 26, 34, 35]. Although longer follow-up times are better to evaluate rate and durability of healing, they are not always feasible. A group of authors frequently working on cartilage healing on dogs recommend minimal evaluation time as 15 weeks. Current study was terminated at 15 weeks. Longer follow-up might result in better healing or degeneration and worsening.

The weakest point of the study was the sample size. Using large animals for the experiment requires larger resources. Both economic and ethical considerations prevented conduction of the study on a larger sample size, and this resulted in difficulties in statistical evaluation.

The MIV technique seems to yield similar results with MACI. In case of its clinical use, it would be possible to treat chondral defects in a single session of surgery and would cost less than MACI. There are other single session choices for cartilage repair. One of the most commonly used ones is to cover the microfracture applied area with a synthetic or collagen membrane [36]. There is literature about both its success and failure, but it still enjoys common use [36, 37]. The next possible step in investigation would be comparing this method with the one we have described.

## Abbreviations

ACI: Autologous chondrocyte implantation
DMEM: Dulbecco’s modified eagle Medium
ELISA: Enzyme-linked immune sorbent assay
GAG: Glycosaminoglycan
GF: Growth factors group
ITS: Insulin-Transferrin-Selenium
MACI: Matrix-induced autologous chondrocyte implantation
MIV: Collagen membrane group
OAT: Osteochondral autogenous transfer
